# A Legion of Leeches

**Published:** 1871-01

**Authors:** 


### A LEGION OF LEECHES.

Seventy-four thousand doctors! Think of it. All this number in our country, according to the present census, unless the newspapers inform us falsely. In 1860 there were 55,000—an increase of 19,000 in ten years, or nearly 2,000 a year!

Ought not these figures to "give us pause?" Reflect a moment what an army they would make, even in this day of big armies; or what a city they would form, larger than any in many of the oldest States.

Or, look at it again from another point of view. What a mint of money it takes to support this army! Probably we are within the mark when we calculate that the average income of the 74,000 from practice is a $1,000 a year each. This makes $74,000,000 a year, which the sick pay for medical advice. For their medicines it is safe to say they pay the odd $26,000,000, which remains to make up $100,000,000 a year, as what sickness costs the American people. And in this calculation we have left, altogether, out of account the tons and hogsheads of quack medicines, which this misguided people pour down their throats. We could safely estimate that at $25,000,000 a year more.

As we are economical in spirit, would it not be well to save some of this? Can it not be done? Let the people study these figures a while, and then reflect that probably one-half, or certainly a large fraction of this expense, is incurred by a deliberate infraction of the laws of health; that if they tippled less, smoked less, overworked less, were less given to lechery and wantonness, ate slower, exercised more judiciously, were less "fast," and less self-indulgent, they would save thirty or forty millions a year. When hygiene is at a loss for any other argument, she can appeal to frugality, and satistics will show that the appeal is a wise one.

Making money is in America the "chief end of man"—as the Westminster catechism has it. Plenty of advisers are ready with their wise saws to tell how it can be accomplished. We are one of them, and offer a saw quite as true and less trite than any of them, and it is this—keep healthy. Living in the midst of a commercial mart, and in the thick of the desperate conflict for wealth, we have seen many a hero in the fight lose all for want of health, lose it, perhaps, just at the moment when a month or two more of work would have made a fortune.

It is said that when Alexander VI died, his son, the famous Cæsar Borgia, had every provision made to seize the supreme power and make himself master of Italy, that he had every possible contingency guarded, but one, and that was his own physical inability to take advantage of the crisis. But sickened to threatening illness, by the same poisoned wine which killed his father, he lost his chance and died defeated, an exile and a captive. It were well if many an American business man took warning by the moral this fragment of history conveys, and would remember that the labor of life may be lost by the preventable illness of a week. —*Medical and Surgical Reporter.*

